# Effect of Pre- and Postprandial Plasma Glucose Levels on Thyroid Hormones: A Cross-Sectional Study

**DOI:** 10.7759/cureus.71756

**Published:** 2024-10-18

**Authors:** Sathiyanarayanan Sathiyamoorthi, Suprabha Sinha, Navya Krishna Naidu, Rajeev Aravindakshan

**Affiliations:** 1 Department of Community and Family Medicine, All India Institute of Medical Sciences (AIIMS) Mangalagiri, Guntur, IND

**Keywords:** blood glucose effect on thyroid hormones, diabetes mellitus, hypothyroidism, prandial glucose, thyroid hormones

## Abstract

Background

There is not much data regarding the effect of plasma glucose on thyroid hormones. Currently, there is no consensus regarding the timing of blood sample collection for thyroid hormones. Evaluation of the correlation between plasma glucose and thyroid hormones may enhance pathophysiological understanding of postprandial thyroid stimulating hormone (TSH) decline.

Objectives

To study the effect of pre-and postprandial plasma glucose levels on thyroid hormones.

Methodology

A cross-sectional study was done among participants aged 18 years and above after obtaining informed consent. Venous blood samples for fasting and postprandial plasma glucose, fasting, and postprandial thyroid profile ((FT3), (FT4), and TSH) were collected. The association was studied using the t-test and chi-square test between groups, while correlation using Pearson’s correlation coefficient. A p-value of <0.05 was considered statistically significant.

Results

Among the 197 participants, 126 (64%) were female and 71 (36%) were male. The mean (± S.D.) of age was 43.74 (± 12.62) years. Mean (± S.D.) postprandial TSH (4.31 μU/ml (± 7.79)) and free T3 (285.52 pg/dl (± 32.46)) were less than the fasting values (4.99 μU/ml (± 7.78)) and (295.84 pg/dl (± 32.56)). The mean (± S.D.) of both fasting and postprandial TSH and free T4 were less in the high plasma glucose group compared to the normal plasma glucose group (fasting state p-values 0.005, <0.0001 & postprandial state p-values 0.016, <0.0001). The correlation between fasting plasma glucose and fasting TSH values (Pearson correlation; r=-0.18; p-value 0.232) was observed across all the plasma glucose ranges.

Conclusion

There is a significant postprandial decline in TSH levels than the fasting TSH, indicating that there is a plasma glucose-mediated reduction in TSH values.

## Introduction

The number of people with diabetes in India, currently around 40.9 million, is expected to rise to 69.9 million by 2025 with continuing trends [1]. Hypothyroidism is the most common thyroid disease, affecting one out of 10 persons in India, with the prevalence of overt and subclinical hypothyroidism at 10.95% and 8.02%, respectively [2]. A higher prevalence of overt hypothyroidism in type 2 diabetes mellitus (T2DM) than in the general population has been noted [3].

Subclinical hypothyroidism (SCH) is associated with insulin resistance, dyslipidemia, hypertension, high cholesterol, low mood, infertility, and cardiovascular comorbidities [4,5]. Early detection of SCH and starting early supplementation will help in improving the quality of life of people having SCH [6]. Serum TSH thus forms the basis for detecting thyroid dysfunction and its management. Thyroid function tests might be discordant due to assay interferences and/or the effects of concurrent medications, pregnancy, critical illness, timing of sample collection, diurnal variation, and most importantly, the fasting/fed status of the patient [7-10].

In routine clinical practice, limited importance is given to the timing of sample collection. It is generally observed that TSH levels in early morning fasting states were higher than TSH levels measured later in the same day [11]. Few studies have demonstrated postprandial decline in TSH [12-14]. Diurnal variations and fed status have been claimed to alter TSH levels, and diurnal variation in TSH is a main confounding factor in the assessment of the effect of the postprandial state on changes in TSH [15-17]. There is a need to add more information about how one’s diabetic status or plasma glucose levels may affect their thyroid hormones, thus enhancing pathophysiological understanding of postprandial TSH decline, which in turn will be helpful in clinical decision-making in the management of thyroid disorders. Therefore, this study aims to study the effect of pre-and postprandial plasma glucose levels with thyroid hormones and their correlation.

## Materials and methods

Study design, participants, inclusion, and exclusion criteria

A comparative cross-sectional study was conducted at All India Institute of Medical Sciences (AIIMS) Mangalagiri, Guntur, India, among both male and female participants aged 18 years and above after obtaining informed consent. Hypothyroid patients on thyroxine supplementation and T2DM patients on anti-diabetic drugs were also included in the study. Participants who were hyperthyroid and had a history of suspected thyroiditis, recent hospitalization for critical illness, patients with renal or liver dysfunction, pregnant women, and use of drugs interfering with thyroid function such as steroids, amiodarone, iodine-containing drugs, and glucocorticoids within the last six months were excluded.

Sample size

Expecting a difference in pre- and post-meal TSH values, the sample size was calculated using nMaster 2.0 (Informer Technologies Inc., Los Angeles, United States) sample size software based on an Indian study [10] with fasting TSH (mean 2.42 ± 1.49) and post-meal TSH (mean 1.79 ± 1.01) with 80% power and 5% α error, which came to 136. Considering 10% non-response, a minimum of 160 participants needs to be studied.

Data collection methods

A basic, pre-tested structured questionnaire was used to collect basic demographic data, including age, gender, address, highest educational qualification, occupation, and socio-economic status [18], complaints if any, and any pre-existing disease, along with the number of years he/she has had it for. Anthropometric measurements were (height and weight) recorded. Venous blood samples for fasting and postprandial plasma glucose and fasting and postprandial thyroid profile ((FT3), (FT4), and TSH) were collected after an overnight fast for at least 10 hours. Samples were analyzed by chemiluminescence immunoassay using the Cobase 411 immunoassay analyzer for thyroid hormone estimation. Reference ranges for TSH, free T4, and free T3 were taken as 0.4-4.2 μU/ml, 0.8-2.7 μg/dl, and 210-440 pg/dl, respectively. Thyroid disorders like SCH, overt hypothyroidism, and hyperthyroidism were defined based on thyroid hormone levels as per standard guidelines [19]. Plasma glucose was estimated using the glucose oxidase peroxidase method and classified according to American Diabetes Association guidelines [20].

Statistical analysis

The data were entered and analyzed using the SPSS for Windows, Version 15.0 (SPSS Inc., Chicago, United States). Categorical data are reported as frequencies and percentages. A paired t-test was done to compare pre-and postprandial levels of thyroid hormones. A paired sample t-test was done to measure the association of continuous variables between the two groups. A chi-square test was done to measure the association of categorical variables between the two groups. A correlation between plasma glucose and thyroid hormones was studied using Pearson’s correlation coefficient. A p-value of <0.05 was considered statistically significant.

Ethics statement

The study was approved by the Institutional Ethics Committee (IEC/2020-21/68) on 03/10/2020. Written informed consent was obtained for participation in the study and use of the patient data for research and educational purposes. The procedures follow the guidelines laid down in the Declaration of Helsinki 2008.

## Results

The study was conducted among 197 participants, and their mean (± S.D.) age was 43.74 (± 12.62), ranging from 18 to 73 years. Out of which, biochemical values were available for about 175 participants. So, further analysis and analysis for statistical associations were restricted to these 175 participants. In the study population, 36 (18.3%) of the study population were illiterate, 44 (22.3%) had studied up to middle school, 52 (26.4%) had studied up to high school, and 11 (5.6%) studied up to higher secondary. About 45 (22.8%) were graduates, while nine (4.6%) had done postgraduation. Baseline characteristics, demography, and morbidity profile are shown in Table 1.

**Table 1:** Socio-demographic, baseline, and morbidity profile of the participants (n=197)

Category	Frequency	Percentage (%)
Gender		
Male	71	36.0
Female	126	64.0
Age groups		
18 – 35 years	57	28.9
36 – 59 years	114	57.9
60 years & above	26	13.2
Nutritional status		
Underweight	05	2.5
Normal	49	24.9
Overweight	76	38.6
Obese	67	34.0
Known case of any chronic disease (DM, hypothyroidism, HTN, etc.,)	151	76.6
Known case of diabetes mellitus	92	46.7
Known case of hypothyroid	63	32.0
Known case of hypertension	37	18.9
Known cases of other chronic diseases	08	4.06
Known cases of both diabetes & hypothyroid	10	5.0

All the study participants were subjected to biochemical investigations: fasting plasma glucose, fasting TSH, fasting free T3, fasting free T4, postprandial plasma glucose, postprandial TSH, postprandial free T3, and postprandial free T4. Participants were classified for diabetes and thyroid status based on the biochemical values, both in fasting and postprandial states, in Table 2.

**Table 2:** Baseline disease status based on test values among the study participants (n=175)

Variable	Fasting		Postprandial	
	Frequency	Percentage	Frequency	Percentage
Plasma glucose				
Normal	41	23.4	63	36.0
Impaired	57	32.6	41	23.5
Diabetes	77	44.0	71	40.6
Thyroid status				
Subclinical hyperthyroidism	04	2.3	06	3.4
Normal	103	58.9	109	62.3
Subclinical hypothyroidism	50	28.6	38	21.7
Primary hypothyroidism	12	6.9	10	5.7
Secondary hypothyroidism	06	3.4	12	6.9

The diabetic status of the study participants was recorded considering both fasting and postprandial plasma glucose levels. It was found that 37 (21.1%) had normal levels, 50 (28.6%) had impaired glucose, and 88 (50.3%) had their plasma glucose in the diabetic range. The mean (± S.D.) postprandial TSH and free T3 were less than the fasting values, and this difference was statistically significant by the paired ‘t’ test in Table 3.

**Table 3:** Comparison of fasting and postprandial thyroid profile (n=175) ^*^statistically significant TSH: thyroid stimulating hormone

Variable	Fasting		Postprandial		p-value
	Mean	S.D	Mean	S.D	
TSH	4.99	7.78	4.31	7.79	<0.0001*
Free T3	295.84	32.56	285.52	32.46	<0.0001*
Free T4	1.03	0.22	1.03	0.19	0.459

To test the effect of plasma glucose on thyroid profile, the mean (± S.D.) of thyroid profiles was compared between two groups: the normal plasma glucose group and the high plasma glucose group; participants with normal and impaired plasma glucose were in the normal plasma glucose group and those with diabetic range were in the high plasma glucose group. Thus, out of 175 participants, 87 (49.7%) had normal/impaired glucose levels, and 88 (50.3%) had glucose levels in the diabetic range. The mean (± S.D.) of TSH was less and free T4 was more in the high plasma glucose group compared to the normal plasma glucose group, and this difference is statistically significant by the independent sample ‘t’ test in Table 4.

**Table 4:** Association between plasma glucose and thyroid profile (n=175) ^*^statistically significant TSH: thyroid stimulating hormone

Thyroid profile	Normal plasma glucose Mean (S.D.)	High plasma glucose Mean (S.D.)	p-value
Fasting TSH	6.65 (10.53)	3.35 (2.45)	0.005*
Fasting free T4	0.97 (0.23)	1.09 (0.18)	<0.0001*
Fasting free T3	297.54 (37.02)	294.15 (27.56)	0.495
Postprandial TSH	5.75 (10.69)	2.88 (2.07)	0.016*
Postprandial free T4	0.97 (0.19)	1.09 (0.17)	<0.0001*
Postprandial free T3	287.48 (35.51)	283.58 (29.21)	0.429
Total	37	138	175

In this study, the participants were classified into two groups based on their fasting plasma glucose as normal/impaired and diabetic plasma glucose. It was found that the proportion of subclinical/overt hypothyroid state present among the diabetes participants was less compared to normal plasma glucose level participants using the chi-square test. This observation was found in both fasting and postprandial samples in Table 5.

**Table 5:** Association between fasting plasma glucose and presence of thyroid disorders among participants (n=175) ^*^statistically significant

Variables	Thyroid status			
	Normal	Subclinical/overt hypothyroidism	Total	P-value
Fasting plasma glucose				
Normal/impaired	53 (54.1)	45 (45.9)	98	0.03*
Diabetes spectrum	54 (70.1)	23 (29.9)	77	
Postprandial glucose				
Normal/impaired	61 (58.7)	43 (41.3)	104	0.01*
Diabetes spectrum	55 (77.5)	16 (22.5)	71	

**Figure 1:**
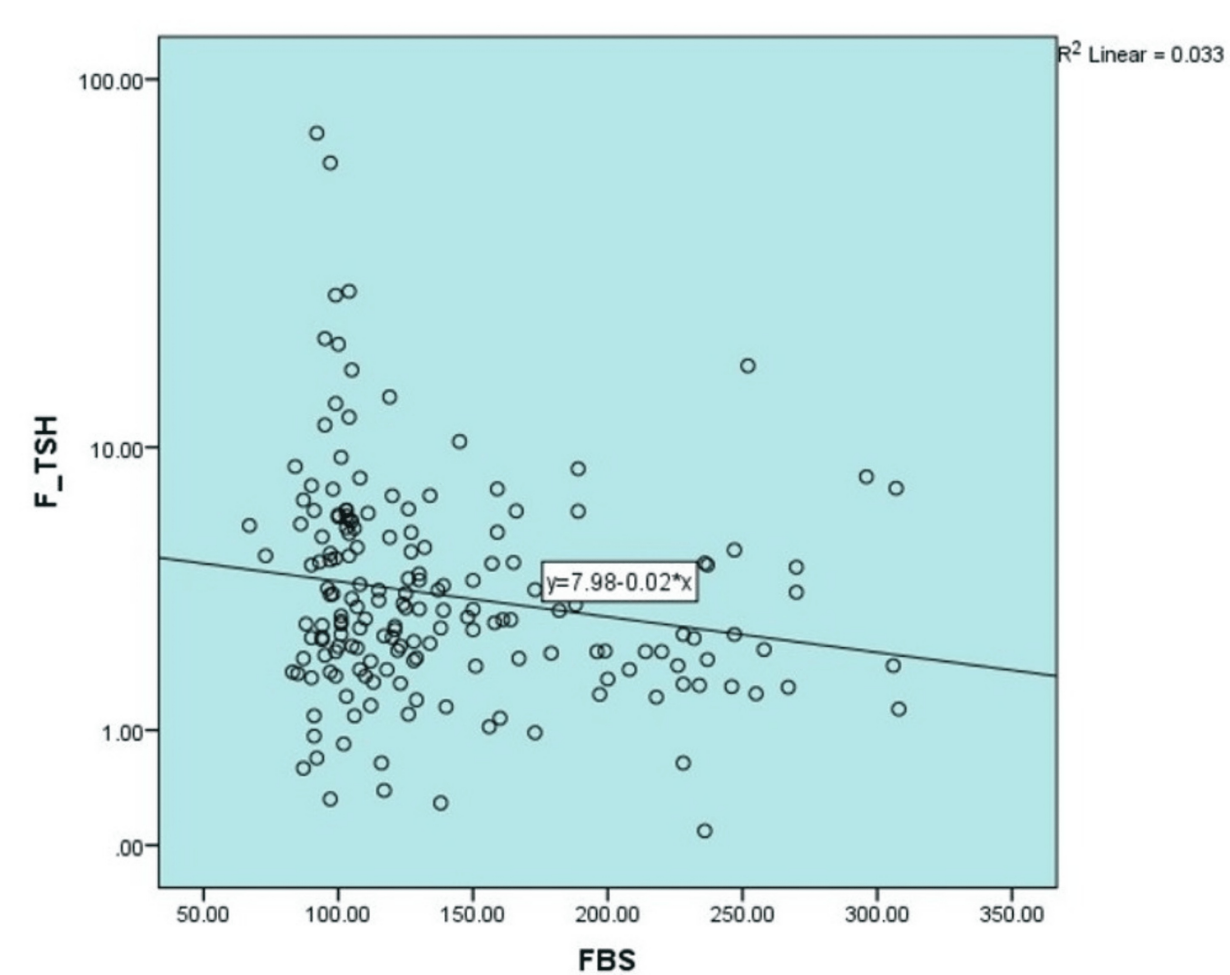
Correlation between fasting plasma glucose and fasting TSH TSH: thyroid stimulating hormone

**Figure 2:**
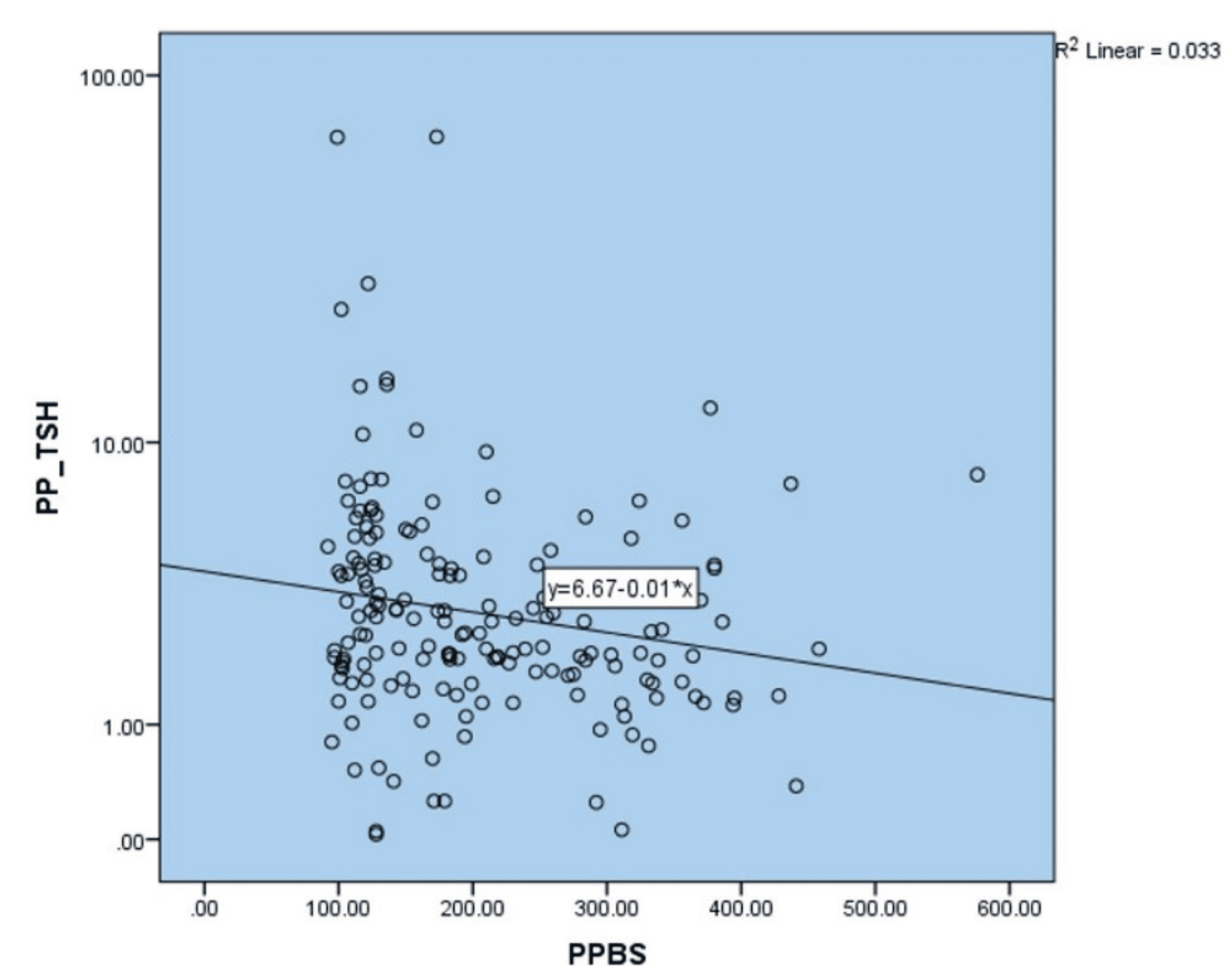
Correlation between postprandial plasma glucose and postprandial TSH TSH: thyroid stimulating hormone

The correlation between fasting plasma glucose and fasting TSH values (Pearson correlation; r=-0.18; p-value 0.232) was 0.18 across all the plasma glucose ranges. A similar effect was noted in postprandial glucose values, also in Figures 1, 2.

## Discussion

The study was conducted to find the effect of pre-and postprandial plasma glucose levels and thyroid hormones. The mean age was 43.74 ± 12.62, which is comparable to the study done by Pradeep et al. [21]. The nutritional status of the participants revealed that 2.5% were underweight, 24.9% had normal weight, 38.6% were overweight, and 34% were obese. Chronic illnesses were present in 76.6% of patients. Only 5.1% of the study participants reported having co-existing hypothyroidism and diabetes. The study found that 88 (50.3%) had their plasma glucose in the diabetic range, as compared to 27% in the study done by Pradeep et al. [21].

Serum TSH was less and free T4 was more in the high plasma glucose group compared to the normal plasma glucose group, and the difference was statistically significant.

There was a significant decline in TSH and free T3 values postprandially, while no such decline was observed in free T4 levels. In a study by Pradeep et al. [21], there was a significant decline in TSH values postprandially, but FT4 didn’t alter significantly [21]. Timing of sampling was considered a factor in the decline in TSH in a previous study [14]. Few studies have shown comparable TSH decline in postprandial TSH fall and extended fasting states, suggesting timing of sample collection is more important than the fasting/fed state [22-25].

In the present study, it was found that the proportion of subclinical/overt hypothyroid state present among the diabetes participants is less compared to normal plasma glucose level participants in both fasting and postprandial samples. This contrasts with a study done by Kaur et al. in which TSH levels were significantly high in T2DM patients [26]. Like the Kaur study, Sotak et al. also found that patients with T2DM showed a higher prevalence of primary hypothyroidism [26,27]. This could be because of other factors like interleukins and cytokines that affect the somatostatin release or affect the TSH directly [28,29].

Among the 96 participants unaware of their thyroid and diabetes status, 73 (76.04%) had normal or impaired plasma glucose levels, while 23 (23.95%) had high plasma glucose levels. Notably, 56.2% of participants with normal plasma glucose had co-existing thyroid disorders, compared to only 30.4% of those with high plasma glucose. This prevalence is significantly higher than the 16.2% reported by Jali et al. among patients with type 2 diabetes mellitus (T2DM) [30]. 

Postprandial TSH values could underdiagnose subclinical hypothyroidism (SCH). Approximately 28.6% of participants were identified as having SCH based on fasting thyroid values, compared to only 21.7% based on postprandial values. This is similar to a study done by Nair et al., which reported a 75% reduction in SCH between pre- and postprandial levels [10]. Our study findings were consistent with the correlation of 0.2 between the absolute postprandial change in plasma glucose and TSH, as reported by Pradeep et al. [21].

High plasma glucose levels were found to affect TSH values in our study. Patients with diabetes and uncontrolled plasma glucose levels can also underdiagnose co-existing thyroid dysfunction. In pregnant women with gestational diabetes mellitus (GDM), it is even more important because high plasma glucose levels might underdiagnose thyroid dysfunction in them [22]. Based on the above observations, fasting and morning plasma samples demonstrate better diagnostic value for thyroid disorders than postprandial or random samples. Revised guidelines should recommend glycemic control for patients with diabetes and high plasma glucose levels, with different reference ranges for thyroid hormones to enable early and accurate detection of thyroid dysfunction. Although the differences between fasting and postprandial values were statistically significant for TSH (4.99 vs. 4.31) and T3 (295.84 vs. 285.52), with a correlation of -0.18 for TSH, these differences may lack clinical relevance. However, there was a significant difference, both clinically and statistically, among patients with poor glycemic control, as suggested by the current study.

Limitations

The study's cross-sectional design limits causality assessment and lacks temporality while confounding factors like diurnal variation and stress were not fully controlled. Excluding certain populations restricts generalizability, and small subgroup sizes may hinder significant association detection. Variations in medication regimens were also not accounted for.

Recommendations

A uniform national guideline may be advised for the appropriate timing for sample collection for thyroid profile. Different reference ranges can be used for thyroid values based on whether the patient is in a fasting or fed state.

## Conclusions

There is a significant postprandial decline in TSH levels than the fasting TSH, and the proportion of subclinical/overt hypothyroid state present among the diabetes participants is less compared to normal plasma glucose level participants, indicating that there is a plasma glucose-mediated reduction in TSH values. Thus, caution should be taken in interpreting the thyroid profile, keeping in account the patient’s fed state.
